# Interactions Between Pathogenic *Burkholderia* and the Complement System: A Review of Potential Immune Evasion Mechanisms

**DOI:** 10.3389/fcimb.2021.701362

**Published:** 2021-09-30

**Authors:** Irum Syed, R. Mark Wooten

**Affiliations:** Department of Medical Microbiology and Immunology, University of Toledo College of Medicine and Life Sciences, Toledo, OH, United States

**Keywords:** *Burkholderia*, melioidosis, glanders, cystic fibrosis, complement, immune evasion, lung infections, virulence mechanisms

## Abstract

The genus *Burkholderia* contains over 80 different Gram-negative species including both plant and human pathogens, the latter of which can be classified into one of two groups: the *Burkholderia pseudomallei* complex (Bpc) or the *Burkholderia cepacia* complex (Bcc). Bpc pathogens *Burkholderia pseudomallei* and *Burkholderia mallei* are highly virulent, and both have considerable potential for use as Tier 1 bioterrorism agents; thus there is great interest in the development of novel vaccines and therapeutics for the prevention and treatment of these infections. While Bcc pathogens *Burkholderia cenocepacia*, *Burkholderia multivorans*, and *Burkholderia cepacia* are not considered bioterror threats, the incredible impact these infections have on the cystic fibrosis community inspires a similar demand for vaccines and therapeutics for the prevention and treatment of these infections as well. Understanding how these pathogens interact with and evade the host immune system will help uncover novel therapeutic targets within these organisms. Given the important role of the complement system in the clearance of bacterial pathogens, this arm of the immune response must be efficiently evaded for successful infection to occur. In this review, we will introduce the *Burkholderia* species to be discussed, followed by a summary of the complement system and known mechanisms by which pathogens interact with this critical system to evade clearance within the host. We will conclude with a review of literature relating to the interactions between the herein discussed *Burkholderia* species and the host complement system, with the goal of highlighting areas in this field that warrant further investigation.

## Burkholderia

The genus *Burkholderia* dates back to the early 1990s, when phylogenetic analysis of 16S rRNA sequences of numerous *Proteobacteria* supported the departure of members of *Pseudomonas* homology group II into a novel genus (Yabuuchi et al., 1992). Named after plant pathologist Walter H. Burkholder, this genus consists of Gram-negative coccobacilli that are ubiquitous within the environment and consists of phytopathogens, as well as obligate and opportunistic mammalian pathogens (Compant et al., 2008).

Within the diverse Gram-negative organisms that comprise the *Burkholderia* spp. are several important human pathogens. A common feature across most virulent *Burkholderia* spp. is the ability to persist both in extracellular spaces and intracellularly within different host cell types, and subsequently evade immune clearance. Thus, they have evolved a large number of strategies to resist antibiotic-mediated effects as well as immune killing mechanisms, including Type III (T3SS) and Type VI (T6SS) secretion systems, actin polymerization, generation of multinucleated giant host cells, and many others. One of the most important host defenses that any intracellular or extracellular pathogen must resist is the host complement system, which is encountered immediately after bacteria enter a vertebrate host. The goal of this review is to discuss the two major groups of *Burkholderia* spp. that cause disease in vertebrate animals and emphasize the data regarding how these pathogens resist the host complement system, identifying gaps in our knowledge that warrant investigation.

## Group I: *Burkholderia pseudomallei* Complex

The *Burkholderia pseudomallei* complex (Bpc) consists of organisms whose genetic content suggests a common ancestral strain similar to *B. pseudomallei*. The best-known members of this group include *B. pseudomallei, B. mallei*, and *B. thailandensis*.

### Burkholderia pseudomallei

*B. pseudomallei* is the causative agent of melioidosis, a disease originally observed by physicians Alfred Whitmore and C.S. Krishnaswami at Rangoon General Hospital in what is now Myanmar (Whitmore and Krishnaswami, 1912). Originally called Whitmore’s disease, this infection was renamed “melioidosis” ten years after its initial discovery. Translated from Greek, melioidosis means “an illness that resembles glanders” and pays homage to the disease it most closely resembles (Whitmore and Krishnaswami, 1912; Stanton and Fletcher, 1921). Melioidosis is a severe febrile disease endemic in tropical and sub-tropical regions, where patients with septicemic melioidosis face a ~40% mortality rate even with antibiotic treatment (White, 2003; Cheng and Currie, 2005). *B. pseudomallei* has been nicknamed “the great mimicker” due to the wide range of signs and symptoms of melioidosis, which often leads to its misdiagnosis and delays appropriate treatment (Yee et al., 1988). Even when positively identified, appropriate treatment of *B. pseudomallei* infections is difficult due to the multitude of antibiotic-resistance mechanisms this pathogen employs. Of note, over half of all melioidosis patients worldwide have either known or undiagnosed diabetes mellitus, making this co-morbidity the most important risk factor for *B. pseudomallei* infections (Wiersinga et al., 2018). As the global prevalence of diabetes continues to rise, the incidence of melioidosis will likely increase as well (Hodgson et al., 2013).

Melioidosis can present in three different disease courses: acute, chronic, or latent. Acute melioidosis is the most common manifestation of this disease, accounting for 85% of cases. Acute melioidosis is characterized by sepsis with or without pneumonia, or the presence of localized abscesses (Currie et al., 2010). Chronic melioidosis makes up 11% of cases and is characterized as a less severe disease with symptoms persisting for over 2 months (Currie et al., 2010). Latent melioidosis cases are rare, comprising only 4% of cases, and is caused by reactivation of *B. pseudomallei* from latent foci from previous infection (Currie et al., 2010). While cutaneous inoculation is the most common route of infection, aerosol delivery of *B. pseudomallei* significantly increases its virulence, with a 99-fold increase in disease potential observed in mice (Warawa, 2010). For this reason, inhalation is considered the most lethal route of infection. While aerosolized *B. pseudomallei* has been recognized for its biological warfare potential, there have been no known intentional exposure events. Regardless, due to the potential for this organism to pose a severe threat to human health and safety, *B. pseudomallei* is listed as a Tier 1 select agent and must be worked with under biosafety level 3 (BSL-3) conditions.

### Burkholderia mallei

*B. mallei* is the causative agent of the disease glanders. Unlike the other *Burkholderia* discussed herein, *B. mallei* is an obligate parasite that is unable to survive in the environment, and is thus not isolated from the soil. Instead, this organism has evolved to persist within more limited animal reservoirs, in particular solipeds such as horses, mules, and donkeys (Van Zandt et al., 2013). Examination of the genomes of Bpc organisms revealed that *B. mallei* is a clone of *B. pseudomallei* that has lost large segments of DNA (Godoy et al., 2003; Ong et al., 2004). This divergence appears to have occurred around 3.5 million years ago and resulted in the loss of genes involved in metabolism (Nierman et al., 2004; Song et al., 2010). This genome reduction pattern is consistent with the fact that *B. mallei* is not well suited to survive in the environment, instead existing as an obligate mammalian pathogen with a restricted host range (Nierman et al., 2004).

The first description of glanders dates back to the third century, when Aristotle wrote “The ass suffers chiefly from one particular disease which they call ‘melis’” (Nierman et al., 2004; Männikkö, 2011). Transmission of *B. mallei* occurs when the bacterium is introduced into a new host, either by inoculation of bacteria below the skin or through contact between infected bodily fluids with mucosal surfaces, such as the eyes, nose, or lungs. In both humans and equids, the course of infection is heavily dependent on the route of transmission. Equine glanders is characterized by the appearance of ulcerative nodules within the body, fever, coughing, depression, and anorexia (Khan et al., 2013). Notably, when equine *B. mallei* infection presents as nodules on the animal surface, the disease is referred to as farcy. In humans, glanders is a febrile illness characterized by ulceration at the site of infection, though localized infections can disseminate throughout the body and cause fatal septicemia (Van Zandt et al., 2013). Notably, human-to-human transmission has never been reported in the United States.

While glanders once affected humans throughout the world, recent technological improvements have decreased our reliance on solipeds for transportation. Testing and euthanasia of animals exhibiting this disease also contributed to the decline in human cases within developed countries. The last naturally occurring case of human glanders in the United States was reported in 1934, and current human cases are sporadic and only occur among those in direct contact with this bacterial isolate or infected animals (Van Zandt et al., 2013). Though rare in developed countries, glanders continues to affect humans and animals in the Middle East, Southeast Asia, Africa, and Australia, and treatment of these infections is hampered by the numerous antibiotic-resistance mechanisms employed by *B. mallei*.

Although human cases have declined considerably, there is still great interest in the development of preventative vaccines and/or effective therapeutic strategies for glanders, as *B. mallei* has an extensive history of use as a bioterrorism agent. Given this organism’s continued potential to pose a severe threat to public health and safety, *B. mallei* accompanies *B. pseudomallei* on the list of Tier 1 select agents, and must also be worked with under BSL-3 conditions.

### Burkholderia thailandensis

When *B. thailandensis* was first isolated from a Thai soil sample, it was believed to be an avirulent, capsule-free *B. pseudomallei* mutant strain. Genotypic and phenotypic analysis of the isolate demonstrated that it was not *B. pseudomallei*, but a unique species altogether, and was named for the country in which it was isolated (Smith et al., 1997). While *B. thailandensis* is considered avirulent in humans, several cases of human infection have been reported, as summarized by Gee and colleagues (Gee et al., 2018). While *B. thailandensis* is readily distinguished from the other Bpc organisms by its ability to assimilate arabinose as a sole-carbon source, the expression of antibiotic-resistance mechanisms shared by other Bpc strains make it no less challenging to manage clinically (Smith et al., 1997; Moore et al., 2004).

While relatively avirulent in humans, *B. thailandensis* causes necrotizing pneumonia in mammalian models of infection, though the dose at which 50% of the animals succumb to the infection (LD_50_) is approximately 10^4^-fold higher than LD_50_ values for either *B. pseudomallei* or *B. mallei* (West et al., 2008; Fisher et al., 2012). While *B. pseudomallei* and *B. mallei* require BSL-3 working conditions for safe handling, the relatively innocuous nature of *B. thailandensis* does not bear such restrictions and is thus approved for use under BSL-2 conditions. Notably, the significant genomic similarity between these strains makes *B. thailandensis* a suitable model for the study of certain *B. pseudomallei*- and *B. mallei*-associated virulence mechanisms without the need for BSL-3 facilities (Haraga et al., 2008).

## Group II: *Burkholderia cepacia* Complex

The *Burkholderia cepacia* complex (Bcc) is a group of over 20 different *Burkholderia* opportunistic pathogens known to cause severe disease in immunocompromised individuals, most notably cystic fibrosis (CF) patients. CF is the most common life-threatening genetic disease among the Caucasian population, affecting approximately 1/2500 children born in this demographic (Welsh et al., 2001). Caused by a mutated cystic fibrosis transmembrane conductance regulator (CFTR) gene, this disease is characterized by the production of a viscous mucus within the lungs which ultimately makes the affected patient particularly vulnerable to respiratory illnesses. While *Pseudomonas aeruginosa* is the most common opportunistic pathogen in the CF lung, Bcc infections are particularly devastating to this population given the severity of the ensuing disease. While chronic colonization with Bcc organisms has little impact on the clinical status of a CF patient, colonization can quickly deteriorate into a systemic infection, called “cepacia syndrome” (LiPuma et al., 2001). Cepacia syndrome is an illness characterized by high fever, necrotizing pneumonia, and an overall unfavorable prognosis (Isles et al., 1984; Mahenthiralingam and Vandamme, 2005). In addition to directly contributing to death of CF patients, those colonized asymptomatically with certain Bcc organisms lose the opportunity to undergo lung transplantation, a well-recognized therapy for patients with end-stage lung disease (Morrell and Pilewski, 2016). While successful transplantation can greatly increase the quality of life and long-term survival of CF patients, those colonized with Bcc pathogens prior to transplantation experience a significantly poorer prognosis than non-colonized CF patients, such that colonized individuals are increasingly considered unfit for transplantation (Snell et al., 1993; LiPuma et al., 2001; De Soyza et al., 2010). Further compounding this issue, CF clinics have been known to experience epidemic spread of transmissible Bcc infections across their patients, who became exposed *via* inadequately-decontaminated equipment or waiting rooms shared between patients (Miyano et al., 2003). Due to the diverse antibiotic and antimicrobial resistance mechanisms employed by Bcc organisms, the best way to approach this spread is patient cohorting. Since these infections were first recognized as transmissible between patients, CF clinics have employed strict policies whereby Bcc-colonized patients remain separated from Bcc-uncolonized patients to prevent intra-clinic spread (Ledson et al., 1998).

The nomenclature of Bcc isolates is complex, as these organisms are indistinguishable by common typing methods such as genomic fingerprinting and PCR (LiPuma et al., 2002; Baldwin et al., 2007). Early studies used “*B. cepacia”* as an umbrella term for these organisms, and care must be taken when reviewing the literature to distinguish between when “*B. cepacia*” is being used as a general term or if that specific species is being discussed (Coenye et al., 2001). Fortunately, these strains have more recently been characterized by their phylogenetic differences into sub-classifications called genomovars (Ursing et al., 1995; Vandamme et al., 1997). The Bcc organisms discussed below include *Burkholderia cenocepacia* (prototypical strain of genomovar III), *Burkholderia multivorans* (genomovar II), and *Burkholderia cepacia* (genomovar I). While the Bcc comprises numerous important pathogens, these organisms have not been studied to the same extent as the Bpc organisms. In addition, the majority of the work performed with Bcc organisms used clinical isolates, which tend to fall under genomovar III, *B. cenocepacia*.

### Burkholderia cenocepacia

*B. cenocepacia* is the Bcc organism most frequently isolated from the CF lung; one analysis of over 600 cases wherein Bcc isolates were recovered from CF patients across the United States found that *B. cenocepacia* was the species recovered in 50% of total cases (LiPuma et al., 2001; Reik et al., 2005). Unfortunately, *B. cenocepacia* is also associated with higher rates of morbidity and mortality among CF patients than other Bcc organisms (LiPuma et al., 2001; Mahenthiralingam et al., 2001). Even asymptomatic colonization with *B. cenocepacia* can have a profound impact on patients’ lives, as chronic *B. cenocepacia* infection is a contraindication for lung transplantation, whereas colonization with other Bcc species does not have this limitation (Snell et al., 1993; De Soyza et al., 2010). Furthermore, members of the *B. cenocepacia* ET12 lineage are recognized as some of the most transmissible Bcc organisms (Johnson et al., 1994). While patients that were not colonized with Bcc organisms were already being kept separate from Bcc-colonized patients in CF clinics, the discovery of transmissible strains strictly of the *B. cenocepacia* species led to additional policies to further separate these patients (Ledson et al., 1998). Identification of conserved markers that can rapidly identify transmissible isolates are needed to help CF clinics efficiently prevent inter-patient spread of these devastating pathogens. While the cable-pilin subunit gene (*cblA*) and *B. cepacia* epidemic strain marker (BCESM) ORF *esmR* were briefly believed to be conserved only in epidemic strains, a subsequent examination into the frequency of these genes disagreed, demonstrating that these genes are not in fact sufficient markers of strain transmissibility (Mahenthiralingam et al., 1997; LiPuma et al., 2001).

### Burkholderia multivorans

*B. multivorans* is the second-most commonly isolated Bcc organism from the CF lung, with a reported rate of 38% (LiPuma et al., 2001; Reik et al., 2005). Unlike most other Bcc isolates, the environmental origin of this organism remains a matter of debate, as this organism is often not isolated from soil samples (Peeters et al., 2016; Tavares et al., 2020). While this organism routinely causes systemic infection in immunocompromised individuals, the morbidity and mortality of *B. multivorans*-caused cepacia syndrome is not as severe as disease caused by *B. cenocepacia* (LiPuma et al., 2001; Mahenthiralingam et al., 2001). While *B. cenocepacia* is the most common Bcc organism transmitted between CF patients, intra-clinic spread of *B. multivorans* has been observed on several occasions as well (Whiteford et al., 1995; Vandamme et al., 1997; Millar-Jones et al., 1998; Mahenthiralingam et al., 2000; LiPuma et al., 2001; Mahenthiralingam et al., 2001). These findings suggest that CF clinics should consider isolating *B. multivorans*-colonized patients the same way as they isolate *B. cenocepacia*-colonized patients.

### Burkholderia cepacia

While *B. cepacia* is the namesake of this group of pathogens, this organism is rarely isolated from humans; an analysis of over 600 CF patients colonized with Bcc isolates found that less than 3% of the patients harbored *B. cepacia* of genomovar I (LiPuma et al., 2001). While *B. cepacia* is generally believed to be less virulent than the other Bcc organisms discussed herein, there has been a single reported case of cepacia syndrome caused by *B. cepacia* (Nash et al., 2011). Notably, in direct contrast to the pattern by which they colonize humans, *B. cepacia* is more readily isolated from the soil than either *B. cenocepacia or B. multivorans* (LiPuma et al., 2001).

## Lipopolysaccharide and Capsule

Bacterial surface components such as lipopolysaccharide (LPS) and capsular polysaccharide have the responsibility of interacting with the outside environment and thus play an important role in protecting bacteria against killing by host immune factors. In particular, both structures are known to modulate the interactions between bacteria and the complement section. Thus, it important that we discuss these structures here, both in general and in *Burkholderia*.

**Figure 1:**
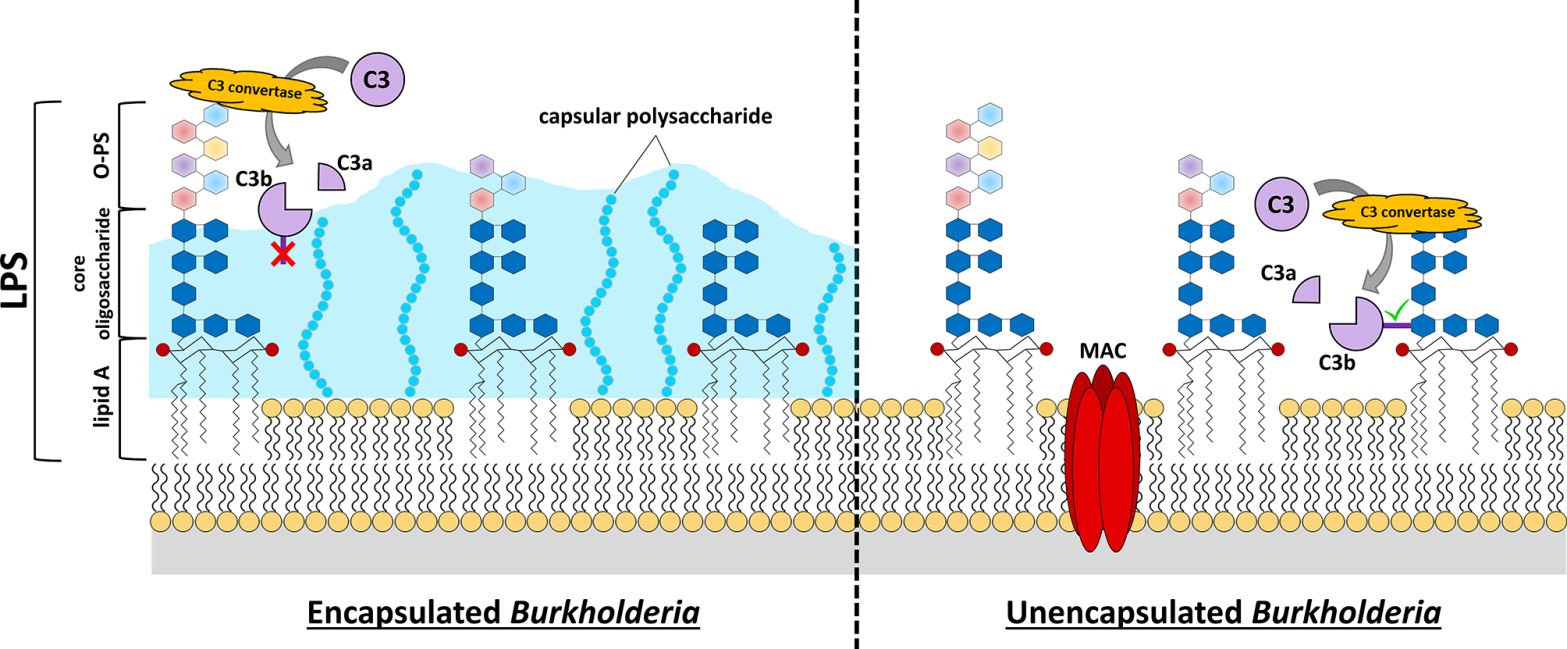
*Burkholderia* cell surface structures involved in evading complement-mediated killing. C3 convertase-mediated cleavage of C3 into C3a and C3b reveals an unstable thioester bond on C3b (dark purple rectangle). If this protein is not generated close to the cell surface or other receptive structures, the thioester bond is quickly hydrolyzed, and the protein loses its enzymatic activity or its ability to attach as an opsonin to the surface (red “X”, C3b in left panel). Many pathogenic *Burkholderia* express capsular polysaccharide that extends quite distant to the cell membrane. This can provide protection from complement activation and opsonization because its structure is not conducive to C3b-binding and, if binding does occur, the bound C3b is relatively distant from the bacterial cell membrane to allow MAC formation. While the lipid A and core oligosaccharide structures of *Burkholderia* LPS are fairly conserved, there is considerable variation in the O-PS of these bacteria (multi-colored hexagons). Analysis of LPS structures of representative *Burkholderia* spp. have demonstrated that these organisms harbor 4-amino-4-deoxy-_L_-arabinose (Ara4N) modifications on the phosphate groups (red circles) of the lipid A disaccharide backbone. The expression of elongated O-PS moieties stretching away from the cell surface can protect against complement-mediated killing by preventing C3 convertase formation close to the cell surface, and thus prevent insertion of a MAC complex in the cell membrane. If C3 convertase forms close to the cell membrane (e.g. LPS core moieties, etc.), the exposed thioester of C3b quickly binds nearby hydroxyl or amino groups (green check mark, C3b in right panel). Complement opsonins that bind distant from the cell surface can promote killing by opsonophagocytosis if they can be recognized by phagocytic cells, however activation/binding near the surface promotes direct killing *via* formation of MAC and bacterial lysis.

LPS is a glycolipid expressed in abundance on the surface of most Gram-negative bacteria that is composed of 3 structural domains: lipid A, core oligosaccharide, and O-antigenic polysaccharide (**Figure 1**) (Raetz and Whitfield, 2002). The acyl chains of lipid A comprise the hydrophobic section of LPS that inserts into the outer leaflet of the outer membrane (Raetz and Whitfield, 2002). Lipid A is often referred to as “endotoxin” because it is held responsible for the toxicity of LPS, however LPS as a whole is sometimes called “endotoxin” as well (Raetz and Whitfield, 2002). The core oligosaccharide is comprised of non-repeating sugar residues that are linked to the membrane-anchored lipid A and extends out, away from the cell surface (Heinrichs et al., 1998). Finally, the O-antigenic polysaccharide (O-PS; also called O-antigen) is composed of a repeating sequence of sugar residues that is attached to the core oligosaccharide and extends further out from the cell (Wang et al., 2010). The O-PS is the most diverse domain of LPS; its composition often differs even within species (Kalynych et al., 2014). Notably, the diverse nature of O-PS structures provides the basis by which Gram-negative bacteria are often classified. Dating back to the 1940s, O-antigen serotyping was used to distinguish strains for clinical and epidemiological purposes (Kauffmann, 1947; Sun et al., 2011).

LPS molecules are tightly packed on the outer membrane surface, with an estimated surface area occupancy of 75% (Lerouge and Vanderleyden, 2002). Given their abundance, LPS are considered essential structural components for most, but not all, Gram-negative bacteria (Zhang et al., 2013). LPS performs numerous important functions, including providing a permeability barrier against small molecules and modulating the host immune response.

The permeability barrier formed by LPS prevents passage of small molecules to the cell surface by both steric and polar means (Bertani and Ruiz, 2018). The ability of extracellular compounds to reach the bacterial surface is physically limited by the presence of abundant tightly-packed LPS molecules (Nikaido, 2003). The assembly of densely-packed LPS molecules is a result of hydrophobic interactions between neighboring lipid A tails; however, this pattern also brings together negatively-charged phosphate groups, which stud the disaccharide backbones of lipid A (**Figure 1**) (Nikaido, 2003). To allow these charged phosphates to exist adjacent to one another, divalent cations (i.e. Ca^2+^, Mg^2+^) are embedded between the LPS molecules to neutralize their overall charge (Nikaido, 2003). The resulting amphipathic environment helps prevent passage of small molecules to the bacterial surface on the basis of polarity (Carpenter et al., 2016).

LPS can also function to modulate the host immune response. While LPS is a pathogen-associated molecular pattern (PAMP) that can robustly induce a pro-inflammatory immune response by activating host toll-like receptor 4 (TLR4), the extensive diversity in LPS structures means that some pathogens express LPS which is less stimulatory than others (Montminy et al., 2006; Bertani and Ruiz, 2018). Furthermore, the expression of LPS – and particularly O-PS – has been shown to prevent complement-mediated cell lysis by preventing the assembly of membrane attack complexes directly on the cell surface (**Figure 1**) (Murray et al., 2006; Goebel et al., 2008; Woodman et al., 2012). In particular, long O-PS chains have displayed an enhanced ability to prevent complement-mediated direct killing in comparison to short O-PS chains, indicating that one mechanism by which LPS prevents serum killing is by physically blocking complement deposition in close proximity to the cell membrane, thus forcing C3 convertases and/or membrane attack complexes to form at a distance away from the bacterial membrane, where they cannot perforate and kill the cell (**Figure 1**) (Kintz et al., 2008).

Another important bacterial surface component is the polysaccharide capsule, which can be expressed by both Gram-positive and Gram-negative bacteria. While not all bacteria express capsular polysaccharide, expression of capsule is an important determinant of virulence, as bacteria that cause invasive disease are often encapsulated (O’Riordan and Lee, 2004). For bacteria that produce a capsule, this structure encases the bacterium and offers protection against environmental stressors as well as effective recognition by the host immune response.

Bacterial capsules are composed of viscous polysaccharides that form a thick layer around the perimeter of the bacterium (Baron, 1996). The hydrophilic nature of capsular polysaccharide provides the bacteria protection against desiccation, allowing for enhanced survival in the outside environment (Angelin and Kavitha, 2020). After entering a host, bacteria must continue to neutralize intra-host environmental stressors, such as antimicrobial peptides. As described above, these compounds kill bacteria by destabilizing the bacterial cell membrane (Bahar and Ren, 2013). Expression of capsule confers protection against antimicrobial peptides by limiting their ability to interact with the bacterial outer membrane surface (Campos et al., 2004).

In addition to protecting against environmental stressors, encapsulated bacteria can modulate the host immune response to survive within the host in ways unencapsulated bacteria cannot. In particular, bacterial capsules often prevent clearance by the host immune response by inhibiting opsonophagocytosis (Domenico et al., 1994; Thakker et al., 1998; Melin et al., 2010; Ali et al., 2019). Opsonophagocytosis is the process by which materials slated for degradation (i.e. microbes, apoptotic host cells) are bound by opsonins, marking them for efficient uptake and clearance by phagocytes. Successful target clearance depends on the interaction between the opsonin and its phagocyte-expressed receptor. The deposition of opsonin within capsular polysaccharide can impede the ability of the opsonin to interact with its cognate host receptor, preventing opsonophagocytosis and allowing for survival of the encapsulated bacterium (Brown et al., 1982; Zaragoza et al., 2003). This mechanism is consistent with the observation that growth of encapsulated bacteria under high-capsule-expressing conditions corresponds to enhanced inhibition of opsonophagocytosis (Nanra et al., 2013). Furthermore, many pathogens have been shown to incorporate host sialic acids into their surface, often becoming an actual part of the capsule (Cress et al., 2014). This activity masquerades the pathogen as a host cell, thus preventing immune activation against the pathogen, as well as the putative ability to bind host complement regulatory proteins.

### Group I: *Burkholderia pseudomallei* Complex

*B. pseudomallei* and *B. mallei* are encapsulated organisms that expresses a number of important virulence factors, including LPS O-antigenic polysaccharide (O-PS) and capsular polysaccharide (**Figure 1**) (Perry et al., 1995; Reckseidler et al., 2001).

The prototypical *B. pseudomallei* LPS O-PS (previously referred to as the *B. pseudomallei* type II O-PS) has the structure -3)-β-_D_-glucopyranose-(1-3)-6-deoxy-α-_L_-talopyranose-(1- (Perry et al., 1995). *B. mallei* is expected to express an identical O-PS, as the *B. mallei* genome contains ORFs identical to those described as the *B. pseudomallei* O-PS biosynthetic gene cluster (DeShazer et al., 1998). This is not surprising, given the clonal nature of these strains (Godoy et al., 2003; Ong et al., 2004). *B. thailandensis*, another highly similar organism, also expresses the *B. pseudomallei* O-PS moiety (Brett et al., 1998). The lipid A disaccharide backbones of these Bpc isolates are often modified with positively-charged 4-amino-4-deoxy-_L_-arabinose (Ara4N) residues. This modification decrease the overall negative charge of the bacteria and protect against interaction with positively-charged antimicrobial peptides or antibiotics (Novem et al., 2009). While not demonstrated in Bpc organisms, expression of the Ara4N biosynthetic cluster is required for the viability of *B. cenocepacia*, and the same may be true for Bpc pathogens (Ortega et al., 2007). While not unique to *Burkholderia*, this modification is not common to all Gram-negative organisms.

The *B. pseudomallei* capsular polysaccharide (previously incorrectly identified and referred to as type I O-PS) is a polymer of 1,3-linked 2-*O*-acetyl-6-deoxy-β-_D_-*manno*-heptopyranose residues (Perry et al., 1995). *B. mallei* capsule is cross-reactive with antiserum against *B. pseudomallei* capsule, however the vast majority of the closely-related *B. thailandensis* strains are unencapsulated and thus do not express this surface structure (Brett et al., 1998; DeShazer et al., 2001). Of note, the *B. thailandensis* variant strain E555 exhibits numerous *B. pseudomallei*-like phenotypes, including expression of capsular polysaccharide nearly identical to that of *B. pseudomallei* (Sim et al., 2010). Importantly, it has been suggested that *B. thailandensis* strain E555 would serve as a better model to study how *B. pseudomallei* interacts with host cells than the more frequently used *B. thailandensis* strain, E264 (Kovacs-Simon et al., 2019).

### Group II:* Burkholderia cepacia* Complex

The O-antigenic polysaccharide (O-PS) of Bcc strains are distinct from those expressed by Bpc strains in that there is no strain-specific consistency among them due to the selective pressure environmental conditions put on the Bcc O-PS gene cluster (**Figure 1**) (Butler et al., 1994; Chung et al., 2003; Hassan et al., 2017; Ruskoski and Champlin, 2017). Strains expressing full-length O-PS are sometimes described in the literature as having “smooth LPS”, and strains expressing truncated or no O-PS are described as having “rough LPS” (Butler et al., 1994). Of note, while strains expressing smooth LPS are more resistant to killing in serum than those expressing rough LPS, there is no association between serum sensitivity and pathogenicity of clinical Bcc isolates (Butler et al., 1994; Zlosnik et al., 2012). This could be attributed to the fact that Bcc pathogens primarily infect immunocompromised populations. Similar to Bpc strains, the lipid A disaccharide backbones of Bcc LPS is modified with cationic Ara4N residues which protect against the activity of antimicrobial peptides (Cox and Wilkinson, 1991; Vinion-Dubiel and Goldberg, 2003; Raetz et al., 2007; Hassan et al., 2017). Notably, the expression of Ara4N biosynthesis enzymes is required for *B. cenocepacia* viability, as they play a role in LPS export to the outer membrane (Ortega et al., 2007; Hamad et al., 2012). These phenomena are unique to *Burkholderia*.

Expression of capsular polysaccharide by Bcc isolates has been suggested to influence strain virulence and, like the expression of O-PS, has been demonstrated to depend on environmental cues (Chung et al., 2003; Ruskoski and Champlin, 2017). Isolates expressing capsule are described in the literature as being “mucoid”, while isolates lacking capsule are described as “non-mucoid” (Cerantola et al., 2000; Ruskoski and Champlin, 2017).

## The Complement System

The complement system is an ancient immune surveillance system and a vital component of the innate immune response (Ricklin et al., 2010). This system is composed of a network of both soluble and membrane-bound proteins which become activated *via* one of three pathways – the classical pathway (CP), the lectin pathway (LP), or the alternative pathway (AP). All of these three activation pathways converge in activation of the C3 component and the subsequent immune effector mechanisms.

**Figure 2:**
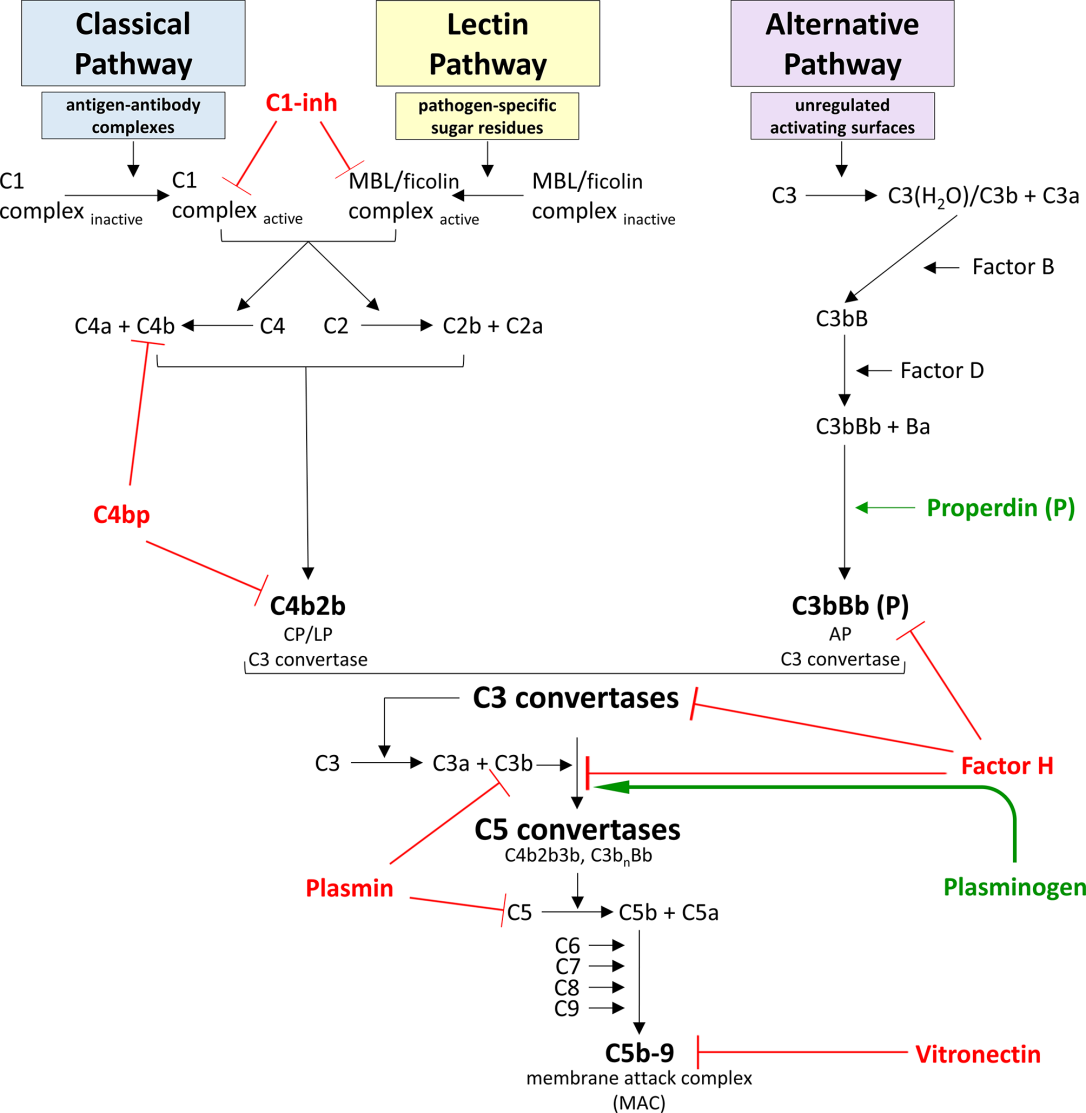
Model for complement system activation and regulation. The complement system is a vital component of the innate immune response and can become activated *via* one of three pathways – the classical pathway (CP), the lectin pathway (LP), or the alternative pathway (AP). The CP is activated when a circulating C1 complex recognizes and binds the Fc region of an antigen-antibody complex. This interaction activates proteases within the C1 complex to cleave C4 into C4a and C4b fragments, and C2 into C2a and C2b fragments. C4b can covalently bind to nearby surfaces, and C2b can bind to the surface-bound C4b to form C4b2b, a C3 convertase. The LP is activated when a circulating mannose-binding lectin (MBL) or ficolin complex recognizes and binds pathogen-specific sugar residues. This interaction activates proteases within the MBL complex to cleave C4 into C4a and C4b fragments, and C2 into C2a and C2b fragments, once again forming C4b2b, the C3 convertase common to both the CP and the LP activation pathways. The AP is unique in that it does not require the presence of specific microbial or “danger” signals to become activated. Instead, the AP maintains a low level of constitutive activation. Successful AP activation occurs when activating surfaces are unregulated, as healthy host cells are protected from complement-deposition by binding regulatory proteins. AP constitutive activation involves spontaneous hydrolysis of C3 into C3a and the C3b-like molecule C3(H2O) in a process called “tick-over”. Factor B binds to C3(H2O), and cleavage of the bound Factor B by Factor D produces C3(H2O)Bb, the soluble C3 convertase of the AP. All C3 convertases cleave C3 into C3a and C3b fragments, and C3b can covalently bind to nearby surfaces. Factor B can bind to surface-bound C3b molecules and be cleaved by Factor D to form C3bBb, the membrane-bound C3 convertase of the AP. This convertase can be bound by properdin to dramatically increase the half-life of this enzyme. Because Factor B can bind to C3b originating from any activation pathway, any C3b bound to a receptive surface will be amplified *via* this AP pathway. The composition of this C3 convertase allows for an efficient cycle of C3b generation and C3 convertase formation that can substantially amplify the complement response irrespective of which pathway initiated the response. The terminal complement cascade is common to all activation pathways and leads to the formation of the membrane attack complex (MAC). In large numbers, MACs can disrupt target cell membranes and cause cell death *via* osmotic lysis and/or loss of membrane integrity. Given the potent pro-inflammatory immune response produced by complement activation, this system must be tightly regulated to prevent unintentional damage to healthy host cells. Regulatory proteins that dampen complement activation and propagation are displayed in red, and regulators that enhance complement are shown in green.

Activation of the CP is initiated when host antibodies bind to an antigen, and this complex is recognized by the complement C1 complex (**Figure 2**). This complex is the Ca^2+^-dependent CP recognition molecule and responds to antigen-antibody complexes. These interactions result in a conformational change that converts the C1 complex from its inactive form to the active form (Roumenina et al., 2005). The activated C1 complex cleaves complement proteins C4 and C2 into C4a + C4b and C2a + C2b, respectively. The larger cleavage products (i.e. “b” fragments) can then come together on cell membranes to form the C3 convertase for the CP, designated C4b2b. It is important to note that the original C2 cleavage product nomenclature was somewhat contentious, but was recently resolved (Bohlson et al., 2019). As such, this convertase may appear as “C4b2a” in some texts.

The LP works similar to the CP and uses much of the same downstream machinery, with the difference being that the LP is initiated by innate recognition receptors rather than antibody complexes (**Figure 2**). Mannose-binding lectin (MBL) is the best-characterized recognition molecule of the LP, but ficolins also serve to that capacity. These molecules circulate in complex with MBL-associated serine proteases (MASPs) in a functionally inactive form. Interaction between these recognition molecules and certain pathogen-specific sugar residue patterns occur in a Ca^2+^-dependent manner and leads to a conformational change that activates the MASPs to cleave complement proteins C4 and C2, and the cleavage products go on to produce a membrane-bound C4b2b C3 convertase, identical to the CP C3 convertase (Teillet et al., 2005).

Unlike the other two pathways, the AP does not require the presence of specific “danger” signals or immune complexes to elicit activation (**Figure 2**). Instead, this pathway monitors for pathogen invasion by maintaining a low level of constitutive C3 activation by spontaneous hydrolysis in a process known as “tick-over” (Lachmann and Halbwachs, 1975; Pangburn et al., 1981). Successful AP activation occurs when activating surfaces are unprotected by complement regulatory proteins (e.g. Factor H) which inhibit subsequent complement component C3 activation. In the absence of this regulation, spontaneous hydrolysis of C3 results in the formation of the intermediate molecule C3(H_2_O) (“C3 water”). This molecule is able to bind complement Factor B in a Mg^2+^-dependent manner, leading to a conformational change on Factor B which exposes a cleavage site for the serine protease Factor D. Proteolytic cleavage of Factor B releases a Ba protein fragment, with the remaining C3(H_2_O)Bb complex acting as the soluble C3 convertase of the AP (Pangburn et al., 1981; Forneris et al., 2010).

Like the membrane-bound C3 convertase enzymes, this fluid-phase C3 convertase can cleave C3 into the anaphylatoxin C3a and an unstable C3b. C3b contains an exposed water-labile thioester group that is rapidly hydrolyzed unless it has been generated close enough to a receptive surface to covalently bind (Law and Dodds, 1997). Given the structural similarity between C3b and C3(H_2_0), Factor B is also able to bind to and undergo Factor D-mediated cleavage upon surface-bound C3b molecules, forming C3bBb, which acts as the membrane-bound C3 convertase of the AP. This convertase can be bound by properdin, the only known positive regulator of the complement system, which functions to dramatically extend the half-life of membrane-bound catalytically-active AP convertases (Fearon and Austen, 1975).

The composition and function of this particular C3 convertase allows for an efficient cycle of C3b generation and C3 convertase formation that can substantially amplify the complement response. Because Factor B is capable of binding to surface-bound C3b regardless of its pathway of origin, all complement pathways are able to amplify the complement response *via* this AP amplification loop (Lachmann, 2009).

### Complement-Mediated Killing

Binding of C3b to the surface of microbes/cells that are unable to prevent this deposition can lead to cell death *via* one of several mechanisms. For the sake of this review, we will focus on the complement-mediated direct- and indirect-killing pathways.

Complement-mediated direct killing involves complement proteins alone. The binding of an additional C3b to an existing C3 convertase results in the formation of a C5 convertase; these complexes are C4b2b3b for the CP and LP, and C3b_n_Bb for AP (**Figure 2**). C5 convertases cleave the soluble complement protein C5 to form a C5a anaphylatoxin and an unstable C5b fragment. If C5b is not stabilized by binding to complement protein C6, the protein is rapidly hydrolyzed. If the binding of C6 to C5b is followed by C7 attachment, the trimolecular complex C5b-6-7 inserts into the target lipid bilayer *via* hydrophobic interactions. This complex acts as a scaffold for the recruitment of complement protein C8, as well as several units of C9, forming a transmembrane pore called a membrane attack complex (MAC) (**Figure 2**) (Podack et al., 1979). In large numbers, MACs disrupt target cell membranes and cause cell death *via* osmotic lysis and/or loss of membrane integrity.

In contrast, complement-mediated indirect killing involves complement proteins working in conjunction with certain immune cells to achieve target cell death *via* opsonophagocytosis. Microbes/cells that cannot downregulate complement activation become opsonized with numerous covalently-bound C3b molecules. Recognition of these opsonins by C3 receptors on phagocytes activates the immune cells to more efficiently phagocytose and kill the target cell. Furthermore, anaphylatoxins C3a and C5a generated during the complement activation processes help recruit immune cells to the target cell to enhance the killing process (Klos et al., 2009).

### Complement Regulation

Due to the potent pro-inflammatory immune responses produced by complement activation, it is important that this system remains tightly regulated to limit activation to the surfaces of pathogens and apoptotic cells, which is essential to prevent unintentional damage to healthy host tissues.

Factor H is the master regulator of the AP, due to its ability to dampen amplification of the complement response (**Figure 2**). In both the fluid phase and on cell surfaces, Factor H downregulates assembly of AP C3 convertases by competing with Factor B for binding of C3b (Kazatchkine et al., 1979). In addition, Factor H acts as a cofactor for Factor I-mediated cleavage of C3b into iC3b, a protein fragment that cannot associate with Factor B and thus cannot contribute to amplification of the complement response (Weiler et al., 1976). Furthermore, Factor H can accelerate the decay of existing C3 convertases to further dampen the complement cascade (Whaley and Ruddy, 1976). Expression of binding sites for polyanionic molecules specifically found on healthy host cell surfaces (e.g. glycosaminoglycans and sialic acids) increases the affinity of Factor H to these cells ten-fold, thus allowing Factor H to specifically protect healthy host tissues (Fearon, 1978; Pangburn and Muller-Eberhard, 1978; Meri and Pangburn, 1990; Ferreira et al., 2010). In the absence of Factor H, unregulated spontaneous activation of complement leads to exhaustion of serum components C3 and Factor B, and is associated with inflammatory diseases such as atypical hemolytic uremic syndrome and membranoproliferative glomerulonephritis (Schreiber et al., 1978; Pickering and Cook, 2011; Roumenina et al., 2011).

The classical and lectin pathways are regulated by C4 binding protein (C4bp) and C1 inhibitor (C1-inh) (**Figure 2**). Like Factor H, C4bp is able to accelerate the decay of the C4b2b C3 convertase (Gigli et al., 1979). Furthermore, C4bp acts as a cofactor for Factor I-mediated degradation of C4b to prevent classical or lectin pathway convertase formation altogether (Fujita et al., 1978). Of note, C4bp can also recognize and mediate Factor I cleavage of C3b for further inhibition of the complement cascade (Fukui et al., 2002). C1-inh is a protease inhibitor that inactivates C1 proteases and MASPs to suppress activation of the CP and LP, respectively (Ziccardi, 1981; Rossi et al., 2001). This inhibitory activity is enhanced by host cell surface receptors such as glycosaminoglycans, allowing for preferential protection of these tissues over non-host cells (Wuillemin et al., 1997).

Additional complement inhibitors include plasmin(ogen) and vitronectin (**Figure 2**). Plasminogen circulates in plasma as an inactive precursor to the proteolytic enzyme plasmin. Plasminogen is able to bind C3b at a unique site, thereby avoiding competition with Factor H for C3b binding (Barthel et al., 2012). Formation of a tripartite complex between plasminogen, Factor H, and C3b enhances Factor H cofactor activity, augmenting Factor I-mediated cleavage of C3b to iC3b (Barthel et al., 2012). What’s more, activated plasmin can directly cleave both C3b and C5 to robustly inhibit the complement cascade in both the fluid phase and on cell surfaces (Barthel et al., 2012). Vitronectin downregulates the assembly of the terminal complement pathway by binding to C8 and preventing formation of the lytic MAC pore (Choi et al., 1989; Preissner et al., 1989).

### Recruitment of Host Complement Regulatory Proteins by Pathogens for Immune Evasion

Many pathogens have evolved mechanisms to escape complement-mediated killing. A recent review examined microbial complement evasion strategies (Meri and Jarva, 2020). A few examples of pathogens that recruit complement regulatory proteins will be discussed briefly herein. However, a more comprehensive list is provided in **Table 1**.

**Table 1:** Abridged list of microbial receptors for complement regulatory proteins and their ligands.

Pathogen	Host Target	Pathogenic Component	References
*Aspergillus fumigatus*	Factor H	AfEno1	(Vogl et al., 2008; Dasari et al., 2019)
	C4bp	AfEno1	(Vogl et al., 2008; Dasari et al., 2019)
	Plasminogen	AfEno1	(Dasari et al., 2019)
*Bordetella pertussis*	Factor H	unknown receptor	(Amdahl et al., 2011)
	C4bp	filamentous hemagglutinin	(Berggard et al., 1997)
	C1-inh	Vag8	(Marr et al., 2007; Marr et al., 2011)
*Borrelia* spp.	Factor H	CRASPs; Erp-family proteins	(Brissette et al., 2009; Lin et al., 2020)
	C4bp	unidentified 43kD protein	(Pietikainen et al., 2010)
	C1-inh	CihC	(Grosskinsky et al., 2010)
	Plasminogen	CRASPs; Erp-family proteins	(Brissette et al., 2009; Hallstrom et al., 2010; Lin et al., 2020; Schmidt et al., 2021)
*Candida albicans*	Factor H	Gpm1	(Meri et al., 2002; Poltermann et al., 2007)
	C4bp	Gpm1	(Meri et al., 2004)
	Plasminogen	Gpm1	(Poltermann et al., 2007)
	Vitronectin	Gpm1	(Poltermann et al., 2007; Lopez et al., 2014)
Dengue Virus	C4bp	NS1	(Avirutnan et al., 2010; Avirutnan et al., 2011)
	Vitronectin	NS1	(Conde et al., 2016)
*Escherichia coli*	C4bp	OmpA	(Prasadarao et al., 2002)
	C1-inh	StcE	(Lathem et al., 2004)
*Haemophilus influenzae*	Factor H	Protein H, P5	(Hallstrom et al., 2008; Fleury et al., 2014; Rosadini et al., 2014; Langereis et al., 2014)
	C4bp	unknown receptor	(Hallstrom et al., 2007)
	Plasminogen	Protein E	(Barthel et al., 2012)
	Vitronectin	*Haemophilus* surface fibrils; Protein E; Protein F; Protein H; P4	(Hallstrom et al., 2006; Singh et al., 2011; Su et al., 2013a; Al-Jubair et al., 2015; Su et al., 2016)
Human Immunodeficiency Virus (HIV)	Factor H	gp120; gp41	(Stoiber et al., 1995a; Stoiber et al., 1995b; Stoiber et al., 1996)
	C4bp	gp120	(Stoiber et al., 1995b)
*Leptospira* spp.	Factor H	Enolase	(Meri et al., 2005; Salazar et al., 2017)
	C4bp	Enolase	(Barbosa et al., 2009; Salazar et al., 2017)
*Moraxella catarrhalis*	Factor H	OlpA	(Bernhard et al., 2014)
	C4bp	UspA	(Nordstrom et al., 2004)
	Plasminogen	UspA	(Singh et al., 2015)
	Vitronectin	UspA	(Su et al., 2013b)
*Neisseria gonorrhoeae*	Factor H	Por1B, LOS	(Ram et al., 1998; Madico et al., 2007)
	C4bp	Por1A	(Ram et al., 2001a; Ram et al., 2001b)
*Neisseria meningitidis*	Factor H	fHbp	(Madico et al., 2006)
	C4bp	PorA	(Jarva et al., 2005)
	Vitronectin	OpcA; Msf	(Griffiths et al., 2011; Hubert et al., 2012)
*Streptococcus pneumoniae*	Factor H	PspC	(Dave et al., 2001)
	C4b	PspA; PspC; LytA; PepO	(Dieudonne-Vatran et al., 2009; Agarwal et al., 2012; Ramos-Sevillano et al., 2015; Haleem et al., 2019)
	Plasminogen	PepO	(Agarwal et al., 2013)
	Vitronectin	Hic	(Kohler et al., 2015)
*Streptococcus pyogenes*	Factor H	M Protein	(Peterson et al., 1979; Horstmann et al., 1988)
	C4bp	M Protein	(Thern et al., 1995)
	Vitronectin	Streptokinase	(Ullberg et al., 1989)
West Nile Virus	Factor H	NS1	(Chung et al., 2006)
	C4bp	NS1	(Avirutnan et al., 2010; Avirutnan et al., 2011)
Yellow Fever Virus	C4bp	NS1	(Avirutnan et al., 2010; Avirutnan et al., 2011)
*Yersinia* spp.	Factor H	YadA; Ail	(Biedzka-Sarek et al., 2008)
	C4bp	YadA; Ail	(Kirjavainen et al., 2008)
	Vitronectin	Ail	(Thomson et al., 2019)

As described above, Factor H is a potent negative regulator of complement activation and amplification. Pathogens that have evolved to recruit Factor H to their surface are protected against complement-mediated killing, ultimately allowing for survival within the host. This protective mechanism was expertly reviewed recently by Ferreira and colleagues and will be briefly described herein (Moore et al., 2021). The first microbe found to bind Factor H as an immune evasion strategy was *Streptococcus pyogenes* (Horstmann et al., 1988). Factor H binding by *S. pyogenes* is primarily mediated by surface-exposed M proteins. Strains expressing M protein variants that are unable to bind Factor H accumulate significantly more complement opsonin C3b on their surface and are more readily phagocytosed than Factor H-binding strains (Peterson et al., 1979; Horstmann et al., 1988). *Neisseria meningitidis* also expresses multiple Factor H-binding proteins (Madico et al., 2006; Lewis et al., 2010; Lewis et al., 2013). fHbp (formerly GNA1870) is the best characterized *N. meningitidis* Factor H-binding protein, and this antigen is a component of two licensed meningococcal vaccines (McNeil et al., 2013; Esposito and Principi, 2014; Gandhi et al., 2016). The ability to usurp host Factor H is not unique to bacterial pathogens. Both the West Nile virus and human immunodeficiency virus (HIV) express proteins that bind Factor H to improve viral survival within the host (Pinter et al., 1995a; Pinter et al., 1995b; Chung et al., 2006). Additionally, the fungal opportunistic pathogen *Candida albicans* binds Factor H in its functionally active form to downregulate complement activation and amplification (Meri et al., 2002). Furthermore, the Factor H-binding ability is not limited to proteins. Many pathogens have evolved to exploit the Factor H inherent ability to bind sialic acid residues on host cells to their advantage by either producing their own sialic acid moieties or incorporating sialic acids from host cells onto the pathogen surface (Ram et al., 1998; Vimr et al., 2004).

Although C4bp cannot impede amplification of the complement response, its ability to downregulate the classical and lectin pathways of complement activation make it an attractive target for recruitment by pathogens. Similar to Factor H-binding, recruitment of C4bp for pathogen survival was first demonstrated with *Streptococcus pyogenes* and is also mediated by surface-exposed M proteins (Thern et al., 1995). The related pathogen *Streptococcus pneumoniae* does not express M proteins, but instead expresses multiple different proteins that bind C4bp for immune evasion (Dieudonne-Vatran et al., 2009; Agarwal et al., 2012; Ramos-Sevillano et al., 2015; Haleem et al., 2019). Virulent *Leptospira* strains can bind C4bp, which corresponds with acquisition of resistance to serum-mediated direct killing, indicating that recruitment of C4bp offers protection against the host immune response and contributes to pathogenicity (Barbosa et al., 2009). Once again, this immune evasion strategy is not limited to bacterial pathogens. A Flavivirus protein common to the important human pathogen dengue virus, West Nile virus, and yellow fever virus directly interacts with C4bp to protect infected cells from complement-mediated lysis (Avirutnan et al., 2011). In addition, opportunistic *Aspergillus* fungal species also bind C4bp for immune evasion (Vogl et al., 2008).

C1-inh can also be appropriated by pathogens to prevent initiation of the classical and lectin pathways. The first report of C1-inh recruitment by a pathogen appeared in 2004, when the ability of the *E. coli* protein StcE to bind C1-inh to host cell surfaces was described (Lathem et al., 2004). The importance of this immune evasion strategy is highlighted by the fact that virulent *Bordetella pertussis* binds C1-inh whereas avirulent *Bordetella* spp. do not (Marr et al., 2007; Marr et al., 2011). Finally, binding of C1-inh to the surface of relapsing fever-causing *Borrelia* spp. significantly enhances survival of these bacteria in serum (Grosskinsky et al., 2010).

Recruitment of plasminogen to the pathogen surface can amplify Factor H cofactor function that augments Factor I-mediated cleavage of the opsonin C3b (Barthel et al., 2012). Furthermore, the bound plasminogen can be activated to plasmin, which further protects the pathogen from the host immune response by cleaving C3b and C5, preventing complement activation in the surrounding area (Barthel et al., 2012). These strategies are utilized by many important pathogens, including *Streptococcus pneumoniae, Haemophilus influenzae*, *Borrelia* spp., and *Moraxella catarrhalis* (Barthel et al., 2012; Agarwal et al., 2013; Singh et al., 2015; Schmidt et al., 2021).

Finally, a growing number of pathogens have been shown to bind vitronectin to their surface, where this regulatory protein continues to prevent complement-mediated direct killing. Pathogens that use this immune evasion strategy include *Streptococcus pneumoniae*, *Haemophilus influenzae*, and *Candida albicans* (Hallstrom et al., 2006; Singh et al., 2011; Su et al., 2013a; Lopez et al., 2014; Al-Jubair et al., 2015; Kohler et al., 2015).

## Burkholderia and Complement

### *Burkholderia pseudomallei *Complex

The vast majority of studies assessing complement evasion by Bpc strains were performed on *B. pseudomallei* strains. *B. pseudomallei* clinical isolates are resistant to killing in normal human serum, indicating they can prevent formation of significant levels of MAC on their surface (Ismail et al., 1988; Egan and Gordon, 1996; DeShazer et al., 1998; Woodman et al., 2012; Mulye et al., 2014). While bacterial capsules are often associated with serum-resistance, capsule-deficient *B. pseudomallei* mutants retain the same serum-resistant phenotype displayed by their wild-type strain (Woodman et al., 2012). Serum-sensitivity assays performed using *B. pseudomallei* strains that are mutated in different outer surface components indicated that the O-antigenic polysaccharide (O-PS) is required for the serum-resistance phenotype (**Figure 1**) (DeShazer et al., 1998; Woodman et al., 2012). This conclusion is supported by the fact that the naturally unencapsulated organism *B. thailandensis*, which expresses the same O-PS as *B. pseudomallei*, is also serum-resistant (DeShazer et al., 1998; Brett et al., 1998; Woodman et al., 2012; Mulye et al., 2014). Notably, while the serum-resistance phenotype of LPS-deficient *B. pseudomallei* is attenuated in comparison to wild-type, this mutant strain is not entirely serum-sensitive (Woodman et al., 2012). These data suggest that the LPS expressed by these *Burkholderia* strains aid in immune evasion by physically preventing deposition of complement proteins directly on the bacterial membrane (**Figure 1**). Evaluation of the serum following incubation with *B. pseudomallei* demonstrated intact hemolytic activity, ruling out failure of complement activation. This finding has been supported more recently in studies demonstrating that *B. pseudomallei* infection causes an upregulation of complement genes in both mouse and non-human primate models (Chin et al., 2010; Ward et al., 2019). Together, these data suggest that an additional, hitherto unknown immune evasion mechanism is contributing to *Burkholderia* serum-resistance (Egan and Gordon, 1996).

While the *B. pseudomallei* capsule is not necessary for serum resistance, it remains an important feature for protection against other complement effector mechanisms. Encapsulated *B. pseudomallei* acquires significantly less C3 opsonin on its surface compared to capsule-deficient *B. pseudomallei* mutant strains, as well as the naturally unencapsulated *B. thailandensis* (Reckseidler-Zenteno et al., 2005; Woodman et al., 2012; Mulye et al., 2014). Quantification of C3 opsonin bound to the surface of *B. thailandensis* variant strain E555, which expresses a *B. pseudomallei*-like capsule, further supports these observations (Sim et al., 2010).

The levels of complement components bound to the surface of Bpc organisms has a dramatic effect on the fate of the bacterium. Even relatively low levels of serum opsonization of *B. pseudomallei* and *B. thailandensis* will enhance bacterial uptake by neutrophils. However, a higher critical threshold of bound complement opsonin is required to promote opsonophagocytic killing of Bcc organisms, and these same levels are required to rapidly trigger robust reactive oxygen species (ROS) production (Egan and Gordon, 1996; Woodman et al., 2012; Mulye et al., 2014). Alternatively, while complement opsonization enhances uptake of *B. pseudomallei* and *B. thailandensis* by macrophages, these phagocytes cannot clear the bacteria unless they have also been pre-activated with IFNγ (Mulye et al., 2014). No work regarding *B. mallei* interaction with neutrophils has been published, but both serum- and antibody-opsonized *B. mallei* are phagocytosed in greater numbers by murine macrophage cell lines than unopsonized *B. mallei* (Whitlock et al., 2008; Whitlock et al., 2009).

The role of opsonizing antibodies in promoting complement-mediated killing of *B. pseudomallei* has also been described. While *Burkholderia*-specific antibodies are not required for complement activation or clearance of *B. pseudomallei*, their presence does enhance neutrophil-mediated killing in a complement-dependent fashion (Egan and Gordon, 1996; Ho et al., 1997). In fact, the presence of *B. pseudomallei*-specific antibodies enhanced complement deposition to the *B. pseudomallei* surface to levels similar to that observed on unencapsulated *B. thailandensis* (Mulye et al., 2014). However, antibodies alone are not sufficient to elicit bacterial direct or opsonophagocytic killing on primary phagocytes (Su et al., 2010; Mulye et al., 2014).

Studies have also compared the relative importance of different complement-activation pathways in depositing complement opsonins on *B. pseudomallei* outer surfaces. Complement activation elicited by *B. pseudomallei* occurs largely *via* the alternative pathway compared to the classical/lectin pathways (Egan and Gordon, 1996; Woodman et al., 2012). *B. pseudomallei* is relatively resistant to alternative pathway-mediated complement opsonization, however serum-sensitive *B. pseudomallei* mutants are killed by mechanisms activated through the alternative pathway (DeShazer et al., 1998; Woodman et al., 2012). Furthermore, opsonizing complement fragments on the *B. pseudomallei* surface were bound to the bacteria covalently *via* the canonical ester linkage (Egan and Gordon, 1996; Woodman et al., 2012).

### *Burkholderia cepacia *Complex

Relatively little work has been performed investigating the role of complement in *B. cepacia* complex (Bcc) infection. While the Bcc group contains important pathogens, *B. cenocepacia* is the most extensively studied member of this group.

Similar to observations with Bpc organisms, the serum-sensitivity profile of Bcc isolates is dependent on both the expression of a bacterial capsule and the LPS O-PS (Butler et al., 1994; Su et al., 2010; Ruskoski and Champlin, 2017). A notable difference, however, is that expression of these virulence factors varies considerably even within each species (**Figure 1**). Indeed, the expression of both capsule and O-PS by Bcc isolates are significantly modulated by changes in the extracellular environment (Su et al., 2010; Ruskoski and Champlin, 2017). While the expression of capsule can influence serum survival, the major determinant of serum resistance in Bcc isolates remains the O-PS. As such, the LPS structure of these organisms has been the subject of more extensive investigation than the bacterial capsule.

While the lipid A core-region is highly conserved between Bcc organisms, the O-PS gene cluster experiences strong selective pressure during chronic infection (Savoia et al., 2008; Hassan et al., 2017). Strains that express intact O-PS are described as having “smooth” LPS, whereas those with truncated or absent O-PS are described as expressing “rough” LPS (Butler et al., 1994). Interestingly, while Butler and colleagues found that strains expressing smooth LPS are generally more resistant to serum-mediated killing than those expressing rough LPS, they could find no particular association between presence or absence of O-PS and the ability of the isolate to infect vulnerable populations (Butler et al., 1994). These findings were later reinforced when Ortega and colleagues found that a defect in O-PS production by *B. cenocepacia* strain K56-2 corresponds to loss of the serum-resistant phenotype (Ortega et al., 2005; Maldonado et al., 2016).

The mechanism behind serum-killing of susceptible Bcc strains has been minimally addressed. Early studies indicated that the bactericidal activity of susceptible isolates is heat-labile, suggesting involvement of the complement system (Butler et al., 1994). Investigation into the involvement of the humoral immune system on serum-mediated killing of Bcc isolates concluded that, while the presence of specific antibody enhanced bactericidal activity against these organisms, the majority of the antibacterial activity in serum relies on the complement system (Butler et al., 1994; Su et al., 2010); this is similar to the observations with Bpc strains.

While the alternative pathway of complement activation is primarily responsible for serum-mediated killing of Bpc organisms, there is no evidence of alternative pathway activation by Bcc isolates (Butler et al., 1994; Ortega et al., 2005; Mulye et al., 2014). Selective inhibition of the classical and lectin pathways by calcium chelation significantly attenuates bacterial killing of susceptible isolates in pooled normal human serum, suggesting a more important role for those pathways for Bcc strains (Butler et al., 1994). Differentiation of whether the classical or the lectin pathway bears greater responsibility for killing of susceptible strains is unclear, as evidence has pointed in both directions. On one hand, the lectin pathway recognition molecule MBL has been shown to bind numerous Bcc clinical isolates and lead to complement activation (Davies et al., 2000). Furthermore, infection with Bcc organisms occurs more frequently in cystic fibrosis patients that carry variant alleles that express structurally abnormal MBL (Garred et al., 1999). On the other hand, a more recent study which utilized classical pathway-deficient C1q-depleted serum demonstrated that bacterial killing of serum-sensitive *B. cenocepacia* strains was dependent on classical pathway activation (Mil-Homens et al., 2014). Overall, it appears that the exact mechanism behind complement-mediated killing of susceptible Bcc isolates warrants further investigation.

## Conclusions and Future Perspective

The genus *Burkholderia* contains many important pathogens that warrant our attention and investigation. A common feature across *Burkholderia* spp. is the ability to persist both extracellularly and within different cell types, all the while evading clearance by the host immune response. Due to the essential nature of complement evasion for microbial persistence, identification of microbial mechanisms for suppressing complement activation and propagation may provide targets for immune-based therapies.

Expression of surface proteins that recruit complement regulators is a well-known mechanism of host immune evasion used by a wide variety of pathogens (**Table 1**). The LPS- and capsule-independent serum resistance phenotypes observed by *Burkholderia* indicates that these bacteria bind one or more complement regulators to evade clearance by the host immune system. In particular, recruitment of Factor H by Bpc organisms is an attractive explanation for the observed resistance to both complement opsonization and complement-mediated direct killing in serum. While binding C4bp, C1-inh, and plamin(ogen) could also explain these phenotypes, the observation that Bpc organisms *B. pseudomallei* and *B. thailandensis* resist complement activation and opsonization *via* the alternative pathway suggests an alternative pathway-specific evasion strategy (Egan and Gordon, 1996; Woodman et al., 2012). While recruitment of vitronectin also contributes to serum resistance of pathogens, its mechanism of action involves specifically inhibiting membrane attack complex formation. While resistance to the action of membrane attack complexes has been demonstrated by *Burkholderia*, there is no evidence that the formation of these lytic complexes is directly inhibited, therefore there is little reason to suspect *Burkholderia* recruit vitronectin as an immune evasion strategy (Woodman et al., 2012). Conversely, Bcc serum resistance appears to have little to do with evading alternative pathway activation. Instead, the classical and lectin pathways have been implicated as important in Bcc pathogen virulence (Butler et al., 1994; Garred et al., 1999; Davies et al., 2000; Mil-Homens et al., 2014). Taken together, these data suggest that complement regulators C4bp and C1-inh may play a more significant role in immune evasion by this subset of *Burkholderia* pathogens.

To capture these extracellular host complement regulators, pathogens express binding proteins on their surface. These exposed binding proteins are susceptible to antibody binding and thus make attractive targets for vaccine development, as has been demonstrated with serogroup B meningococci (MenB) (Meri et al., 2008). Most *Neisseria meningitidis* vaccines are developed against the capsular polysaccharide of each *N. meningitidis* serogroup; however, the MenB capsule possesses similar sugar moieties as those found on the surface of human cells to be sufficiently antigenic (Finne et al., 1983). Rather than targeting the capsular polysaccharide, currently available MenB vaccines instead target the Factor H-binding protein fHbp (Gorringe and Pajon, 2012; Shirley and Taha, 2018). Incidentally, due to the highly conserved nature of this Factor H-binding protein, these vaccines appear to have induced cross-protection against the closely related species *Neisseria gonorrhoeae* (Azze, 2019). These findings indicate that targeting *Burkholderia* proteins that bind complement regulators may not only serve as therapeutic targets, but that such a vaccine may be capable of protecting against more than one of these closely related pathogens.

An additional therapeutic approach that involves preventing complement regulator recruitment by pathogens was first considered a decade ago and appears to be gaining traction in recent years. Chimeric proteins were constructed in which the common microbial binding sites of Factor H (domains 6-7 and 18-20) were fused to the Fc receptors of immunoglobulin (Shaughnessy et al., 2011). The binding of FH18-20/Fc to serum-resistant *N. gonorrhoeae* was found to render many pathogenic strains serum-sensitive. Furthermore, binding of this chimeric protein was observed to enhance complement opsonization to the surface of these bacteria (Shaughnessy et al., 2016). Finally, application of FH18-20/Fc significantly attenuated gonococcal infection in the mouse vaginal colonization model (Shaughnessy et al., 2016; Shaughnessy et al., 2020). Importantly, the observed therapeutic benefits of Factor H-Fc chimeras are not limited to *Neisseria*; binding of FH6-7/Fc resulted in increased complement opsonization and serum sensitivity of non-typeable *H. influenzae* (Wong et al., 2016). Furthermore, the utility of these chimeric proteins as therapies against additional Factor H-binding pathogens is currently being evaluated. These findings indicate that, if *Burkholderia* pathogens also bind Factor H, these immunotherapeutics may also prove useful for the treatment of these important diseases.

As the interactions between *Burkholderia* and the complement system remain poorly studied, investigation into complement regulatory protein recruitment mechanisms employed by *Burkholderia* pathogens warrants further investigation. Understanding these host-pathogen interactions will be key for the development of novel therapeutics against these important pathogens.

## Author Contributions

IS wrote this manuscript and RW contributed to organize and edit the manuscript. All authors contributed to the article and approved the submitted version.

## Funding

This work was supported by National Institute of Allergy and Infectious Disease R01AI121970 (RW).

## Conflict of Interest

The authors declare that the research was conducted in the absence of any commercial or financial relationships that could be construed as a potential conflict of interest.

## Publisher’s Note

All claims expressed in this article are solely those of the authors and do not necessarily represent those of their affiliated organizations, or those of the publisher, the editors and the reviewers. Any product that may be evaluated in this article, or claim that may be made by its manufacturer, is not guaranteed or endorsed by the publisher.
